# EDEM2 is a diagnostic and prognostic biomarker and associated with immune infiltration in glioma: A comprehensive analysis

**DOI:** 10.3389/fonc.2022.1054012

**Published:** 2023-01-16

**Authors:** Yuxi Wu, Haofei Wang, Wei Xiang, Dongye Yi

**Affiliations:** Department of Neurosurgery, Union Hospital, Tongji Medical College, Huazhong University of Science and Technology, Wuhan, China

**Keywords:** EDEM2, glioma, immune infiltrates, immunotherapy, biomarker, drug prediction

## Abstract

Glioma is a highly common pathological brain tumor. Misfolded protein response, which is strongly associated with the growth of cancerous tumors, is mediated by the gene, endoplasmic reticulum degradation-enhancing alpha-mannosidase-like protein 2. However, this gene has not been linked to glioma. To assess the same, we used The Cancer Genome Atlas, Chinese Glioma Genome Atlas, and Genotype-Tissue Expression datasets. The gene was overexpressed in gliomas. This overexpression was linked to unfavorable clinical characteristics, such as the World Health Organization grade, isocitrate dehydrogenase mutation, and the combined loss of the short arm chromosome 1 and the long arm of chromosome 19. Quantitative polymerase chain reaction experiments and immunohistochemistry on clinical samples from our institution verified the gene’s expression and clinical importance. The Human Protein Atlas website verified the messenger ribonucleic acid expression of the gene in glioma cell lines, and immunohistochemistry verified the presence of its protein. A previous survival study indicated that its high expression is substantially related to a bad prognosis. It was identified as an independent predictor of primary glioma prognosis using multivariate Cox regression analysis. To forecast individual survival, we created a nomogram based on this (concordance-index = 0.847). Additionally, functional annotation demonstrated its major role in the control of the extracellular matrix and immune system. The scratch assay and transwell migration assay confirmed the decreased invasive ability of U251 glioma cells with the gene knockdown. Its increased expression was found to be related to the extent of macrophage infiltration using the CIBERSORT, ESTIMATE, Single-sample Gene Set Enrichment Analysis, and Tumor Immune Single-Cell Hub (TISCH) algorithms. The Tumor Immune Dysfunction and Exclusion algorithm revealed that the gene can accurately predict the response of immunotherapy (area under the receiver operating characteristic curve = 0.857). Further, isocitrate dehydrogenase 1 mutation is typically more frequent when the gene expression is high. Finally, five medicines targeting this gene were discovered utilizing the molecular docking program and drug sensitivity analysis of the RNAactDrug website. Low expression of the gene inhibited glioma cell invasion. Therefore, the gene is helpful for the diagnosis, prognosis, and case-specific immunotherapy of glioma.

## 1 Introduction

Glioma is the most common neuroepithelial tumor that occurs in the central nervous system (CNS) (1). Even after surgical resection and chemoradiotherapy, the median survival period for individuals with the most aggressive type of glioblastoma (GBM) is merely 14 months (2). Lower-grade gliomas (LGGs) are the most latent antecedents of GBM (2). Patients with LGG have a survival rate of 5.6–13.3 years, and although it has the potential to progress into GBM, the outlook regarding this is generally positive (1, 3). Targeted treatment for gliomas is currently fraught with formidable difficulties (3). Thus, a proper investigation of glioma-related molecular markers as well as possible targets for customized treatment is necessary.

Owing to the tumor-suppressing mechanisms of the host, the body’s protein synthesis increases and undergoes multiple metabolic modifications compared to normal cells (4). The endoplasmic reticulum (ER) is where they are mostly found, and these ensure that proteins are processed and folded correctly. However, because cancer cells are continually exposed to several stressors both within and outside the cell, there is a significant danger of protein misfolding (4). By preventing the accumulation of unfolded or misfolded polypeptides, the ER-associated degradation (ERAD) mechanism maintains ER proteostasis (5, 6). The unfolded protein response (UPR), which occurs when ERAD is defective, is characterized by the accumulation of many unfolded or misfolded proteins in the ER and the integration of signal transduction pathways to restore ER homeostasis (7–10). As shown by numerous studies, three ER stress sensors, including the protein kinase ribonucleic acid-activated-like ER kinase (PERK), inositol requiring enzyme 1 (IRE1), and activating transcription factor 6 (ATF6), play a significant role in the regulation of glioma proliferation, invasion, epithelial-to-mesenchymal transition (EMT), and chemoresistance (11, 12). Numerous studies have suggested that ER degradation-enhancing alpha-mannosidase-like protein 2 (EDEM2) is involved in the selection and degradation of misfolded glycoproteins. In overexpression experiments, it specifically associates with calnexin (CANX) and thioredoxin domain-containing protein 11 (TXNDC11) to speed up the degradation of typical misfolded glycoproteins (12, 13). In melanoma, EDEM2 has been shown to regulate integrin-1, and protocadherin 2 degradation and trafficking have been linked to melanoma metastasis and invasion (12, 13). However, the specific molecular link between EDEM2 and the UPR within the tumor microenvironment (TME) remains unexplained.

Therefore, we gathered voluminous glioma-related data to examine the role of EDEM2 in glioma prognosis. The potential pathophysiological relevance of EDEM2 was demonstrated by enrichment analysis, molecular interaction network analysis, immune infiltration correlation analysis, and gene mutation analysis, which were all carried out using various R packages. To further individualize prognosis prediction, we developed a nomogram combining clinical signs and EDEM2 expression grouping.

## 2 Materials and methods

### 2.1 Data collection

The Glioma project (670 samples), containing clinical information and gene expression data, was downloaded from The Cancer Genome Atlas (TCGA) database. Duplicate samples were excluded. Ribonucleic acid sequencing (RNA-seq) data were transformed from fragments per kilobase per million (FPKM) to transcripts per million reads (TPM) and log2(x+1). To gain further insight, the samples were divided into two groups according to the median EDEM2 gene expression. Additionally, we collected the Chinese Glioma Genome Atlas (CGGA) cohort, including CGGA_693 and CGGA_325 (http://www.cgga.org.cn/) as a validation set. Since there are some recurrent samples in the CGGA cohort, such samples should ideally be eliminated to increase the cohort’s comparability. We also obtained RNA-seq data from the Genotype-Tissue Expression (GTEx; https://gtexportal.org/home/) for 1152 normal brain tissues. Twenty-six clinical specimens of glioma patients were collected between October 2021 and May 2022 at the Department of Neurosurgery, Wuhan Union Hospital. The ethics committee of the Faculty of Medicine (No. S101/2021) approved this study. All study patients provided their written informed consent for using their tumor tissue samples. **Supplementary Table 2** presents the main clinical information of these samples.

### 2.2 Analysis of survival and expression

GlioVis (http://gliovis.bioinfo.cnio.es/), an online data site for exploring glioma expression datasets as well as the CGGA (Primary), Rembrandt, Gravendeel, Freije, and TCGA-GBMLGG datasets were used by this study to explore the expression and prognostic significance of EDEM2 in glioma patients. R software was used to extract the corresponding normal tissue data from TCGA-GBMLGG and GTEx and compare the expression of EDEM2 between glioma and normal tissues. Receiver operating characteristic (ROC) curves were used to evaluate the diagnostic efficacy of EDEM2 in predicting pathological factors. The expression of EDEM2 in tissues and tumor cells was verified using the Human Protein Atlas (HPA) website (https://www.proteinatlas.org/) (14). Meanwhile, the R software helped analyze the differential expression of EDEM2 in different pathological conditions and draw subgroup Kaplan-Meier survival curves. Additionally, we analyzed the expression and prognostic value of EDEM2 for a wide range of cancers by combining TCGA and GTEx.

### 2.3 Cox regression analysis

To ascertain the impact of EDEM2 expression in glioma patients, we used the univariate Cox regression analysis to compute the relationship between EDEM2 expression levels and the patient cohort. Subsequently, using multivariate analysis, we investigated whether EDEM2 is a standalone predictive factor for survival in glioma patients.

### 2.4 Design and evaluation of nomograms

To individualize the expected survival at one, three, and five years, a nomogram was developed using the independent prognostic variables derived from the multivariate analysis based on the Cox regression model. Using the regression modeling strategies (RMS) program, nomograms were produced (Version: 5.1-4). Plotting calibration curves helped evaluate the graphs, with the 45°line being the greatest predicted value. The nomogram was tested based on the proportional hazard assumption. The concordance index (C-index) was used to compare the nomogram’s and the individual prognostic variables’ predictive efficacy. Additionally, the predictive value was assessed using the time-dependent ROC curve.

### 2.5 Differential gene analysis

Utilizing the R language-related tool, differential gene expression analysis based on the negative binomial distribution (DESeq2), expression differences (high-throughput sequencing counts) between patients with high and low EDEM2 levels were compared to a search for differentially expressed genes (DEGs). Differences were deemed to be DEGs if their log2 fold change (FC) was more than 1 and their adjusted p-value was less than 0.05. The differential sequencing map showed the DEGs on both sides. After loading EDEM2 into the online program called STRING, which gathers a significant quantity of integrated protein interaction data and allows the retrieval of interacting proteins, we could determine the protein-protein interaction (PPI) network information. Significant results had a confidence level greater than 0.4.

### 2.6 Analysis of functional enrichment

The R packages “clusterProfiler” and “org.Hs.eg.db” were used to perform the Gene Ontology (GO) and Kyoto Encyclopedia of Genes and Genomes (KEGG) functional enrichment analyses of the detected DEGs between various EDEM2 expression groups. According to GO and KEGG enrichment studies, DEGs are engaged in several biological processes (BPs), cellular components (CCs), molecular functions (MFs), and in bringing about changes in metabolic pathways. To anticipate EDEM2-associated symptoms and signaling pathways, the Gene Set Enrichment Analysis (GSEA) tool was utilized to assess the differences in signaling pathways between the two groups. To find significantly changed pathways, we ran 1000 duplicate gene set permutation tests for each study. The expression level of EDEM2 was considered as the phenotypic distinguishing factor.

### 2.7 Immune cell infiltration investigation

The Single-sample Gene Set Enrichment Analysis (ssGSEA) algorithm in R was used to examine gene expression levels from published signature gene lists and quantify the relative tumor infiltration levels of 24 immune cell types. To investigate immune cell infiltration levels and the relationship between various expression groups, Wilcoxon rank-sum tests were conducted. We concurrently calculated the makeup of 22 immune cells using the CIBERSORT method (https://cibersort.stanford.edu/) (15). Additionally, using our expression profile and the R software’s ESTIMATE package, an immune infiltration cell score was computed for each sample (16). The Tumor Immune Single-Cell Hub (TISCH) database (http://tisch.comp-genomics.org) helped identify the expression distribution of EDEM2 at the single-cell level to gather more proof supporting the role of EDEM2. Finally, using the Tumor Immune Dysfunction and Exclusion (TIDE; http://tide.dfci.harvard.edu) algorithm, the possible immune checkpoint inhibitor (ICI) responses were anticipated (17).

### 2.8 Analysis of mutational landscape differences

For all TCGA-LGGGBM samples that had been MuTect2 software-processed, we obtained the simple nucleotide variation dataset at level four from the Genomic Data Commons portal (https://portal.gdc.cancer.gov/). We used the sample mutation data along with the data from the “maftools” R software to determine the domain information of the proteins. We utilized the chi-squared test to evaluate variations in gene mutation frequency in each set of samples and presented them using waterfall charts. Exome sequencing data are accessible in TCGA database, where the glioma dataset has 662 samples with mutations. The tumor mutational burden (TMB) is the sum of all nonsynonymous alterations per megabase of genomic sequence, including somatic, coding, and censored mutations as well as base substitutions. The somatic variant data were represented using a mutation annotation format. Following that, we used the “maftools” R package to assess differences between the two groups’ somatic variant data (18).

### 2.9 Sample RNA isolation and quantitative real-time polymerase chain reaction

The tumor tissues of 26 glioma patients who underwent therapy at our facility were processed using the TRIzol reagent to extract the total RNA (Invitrogen, Carlsbad, CA, USA). An automated reverse transcription kit for quantitative polymerase chain reaction (qPCR) was used to create complementary deoxyribonucleic acid (cDNA) according to the manufacturer’s instructions (Vazyme R323-01). Utilizing PCR Bio-rad CFX (Bio-Rad Laboratories, California, USA) and AceQ^®^qPCR SYBR Green Master Mix (Vazyme Q111-02), we detected the qRT-PCR experiments. Using the 2^–ΔΔCt^ method, relative levels of EDEM2 mRNA were normalized to the expression of glyceraldehyde-3-phosphate dehydrogenase (GAPDH) as an internal control. Servicebio Biological Engineering Co. Ltd. chemically created each primer that was utilized (Wuhan, China). The DNA primers specific for EDEM2 and GAPDH amplification were as follows: EDEM2 forward primer 5’-CAGATCCCGCCCACTACAGTT-3’, EDEM2 reverse primer 5’-CTGTCCTGGAGCACTTCAACC-3’; GAPDH forward primer 5’-GGAAGCTTGTCATCAATGGAAATC-3’, GAPDH reverse primer 5’-TGATGACCCTTTTGGCTCCC-3’.

### 2.10 Cell culture and transfection

U251 cell lines were bought from Procell (Wuhan, China). The cells were grown in the Dulbecco’s Modified Eagle Medium (DMEM) with 10% fetal bovine serum (FBS) under ideal circumstances (37°C; 5% carbon dioxide, i.e., CO_2_). Small interfering RNAs (siRNAs) were obtained from Genechem Co., Ltd. (Wuhan, China). Their sequences for EDEM2 are 5’-GCCAUAUGGAACAGUGAACUU-3’ and 5’-AAGUUCACUGUUCCAUAUGGC-3’. They were transfected into cells using Lipofectamine 3000 (Invitrogen) based on the manufacturer’s protocol.

### 2.11 Wound healing and transwell assay

Transfected cells were seeded in six-well plates. A sterile pipette tip was used to make a scratch on the cell monolayer when the cell density reached 100%. After the cells had been cultivated in serum-free media for 24 hours, photographs were taken under an inverted microscope in the same location. In the transwell assay, transwell chambers (Corning, USA), precoated with Matrigel (R&D, USA), were used to analyze the invasive ability of the cells. In the upper compartment, a serum-free medium was used to seed the transfected cells, while a serum-containing medium was supplied to the bottom chamber. The cells were fixed and stained with crystal violet after being incubated at 37°C for 24 hours. Subsequently, the cells were counted using a microscope.

### 2.12 Western blot analysis

The cells were lysed in radioimmunoprecipitation (RIPA) buffer; thereafter, protein samples were extracted and quantified using the bicinchoninic acid (BCA) protein assay kit. Protein mixed with loading buffer was separated using sodium dodecyl-sulfate polyacrylamide gel electrophoresis (SDS-PAGE). Subsequently, the transferred poly(vinylidene fluoride) (PVDF) membrane was blocked using five percent skimmed milk at four degrees centigrade for one hour. The primary antibody (EDEM2, Atlas Antibodies, Sweden; GAPDH, Beyotime, China) was incubated overnight and washed thrice with Tris-buffered saline with 0.1% Tween^®^ 20 detergent (TBST). The PVDF membrane was again washed thrice after being incubated with horseradish peroxidase (HPR)-labeled secondary antibody. The blots were visualized by a Western blot detection system.

### 2.13 Immunohistochemistry

The tissues were embedded in paraffin after being fixed in four percent paraformaldehyde and cut into slices. The slices were treated in gradient hydration, followed by 3% hydrogen peroxide (H_2_O_2_) for 10 min and 1% bovine serum albumin (BSA) for 1 hour. The samples were then incubated with primary antibodies (EDEM2, Atlas Antibodies, Sweden; CD68, Servicebio, China; CD4, Servicebio, China; CD8, Servicebio, China). We used 3, 3’-diaminobenzidine (DAB) staining to detect the signals, followed by hematoxylin counterstaining. Images were taken using the Olympus BX51 microscope (Olympus).

### 2.14 Drug sensitivity and docking studies

A thorough analytic tool for massive pharmacogenomic collections (GDSC, CellMiner ligand-receptor, and CCLE), RNAactDrug, allows users to find relationships between drug sensitivity and EDEM2 molecules (19). The Autodock 4.2 program, which was used to confirm the findings of the screening by docking the active compounds to the EDEM2 protein, was utilized to identify drugs with a certain relevance as candidates for future research. The PyMOL software version 2.0.6 (Schrödinger, LLC) was used to produce the molecular docking data between active compounds and proteins. Thereafter, the Vina script was run to carry out molecular binding energy calculation as well as molecular docking results display. Vina’s binding energy of ≤−5.0 kcal·mol^-1^ and the root mean square deviation (RMSD) value of < 2.00 indicated that both had formed stable docking. Finally, the PyMOL software was used for a three-dimensional (3D) display of the ligand-receptor complex generated from molecular docking.

### 2.15 Statistical analysis

All statistical tests were two-tailed, and the level of statistical difference was set at 0.05. R software (version 4.0.2) was applied for statistical analysis and graphical visualization. Two or more groups of continuous variables were analyzed by the student t-test or Kruskal-Wallis test, respectively. Categorical variables were compared between the groups using the chi-squared test. The Kaplan-Meier curves were analyzed using the log-rank test. Spearman’s correlation analysis assessed the correlation between TMB and EDEM2.

## 3 Results

### 3.1 Patient clinical characteristics

All patients were split into high- and low-expression groups. According to **Table 1**, there was a substantial correlation between EDEM2 expression and isocitrate dehydrogenase (IDH) status, combined loss of the short arm chromosome 1 (i.e., 1p) and the long arm of chromosome 19 (i.e., 19q), i.e., 1p/19q codeletion; age, World Health Organization (WHO) grade, main therapeutic result, and histological type. Gender was not significantly correlated (p = 0.584). The validation dataset also revealed consistent findings for IDH status, 1p/19q codeletion, WHO grade, age, and gender (p = 0.516).

**Table 1:** Clinicopathological features of the two groups in the TCGA and CGGA cohorts.

Characteristic	Low expression of EDEM2	High expression of EDEM2	p
N (TCGA)	335	335	
WHO grade, n (%)			< 0.001
G2	176 (28.7%)	40 (6.5%)	
G3	118 (19.2%)	119 (19.4%)	
G4	4 (0.7%)	156 (25.4%)	
IDH status, n (%)			< 0.001
WT	25 (3.8%)	212 (32.1%)	
Mut	307 (46.4%)	117 (17.7%)	
1p/19q codeletion, n (%)			< 0.001
codel	126 (19%)	42 (6.3%)	
non-codel	209 (31.5%)	287 (43.2%)	
Gender, n (%)			0.584
Female	146 (21.8%)	138 (20.6%)	
Male	189 (28.2%)	197 (29.4%)	
Age, n (%)			< 0.001
<=60	300 (44.8%)	231 (34.5%)	
>60	35 (5.2%)	104 (15.5%)	
Histological type, n (%)			< 0.001
Astrocytoma	104 (15.5%)	88 (13.1%)	
Glioblastoma	4 (0.6%)	156 (23.3%)	
Oligoastrocytoma	92 (13.7%)	36 (5.4%)	
Oligodendroglioma	135 (20.1%)	55 (8.2%)	
Age, median (IQR)	39 (32, 49)	54 (40, 63)	< 0.001
N (CGGA)	313	313	
Gender, n (%)			0.516
Female	133 (21.2%)	124 (19.8%)	
Male	180 (28.8%)	189 (30.2%)	
Grade, n (%)			< 0.001
WHO II	159 (25.4%)	61 (9.7%)	
WHO III	103 (16.5%)	85 (13.6%)	
WHO IV	51 (8.1%)	167 (26.7%)	
IDH mutation status, n (%)			< 0.001
Mutant	209 (35.5%)	100 (17%)	
Wildtype	89 (15.1%)	190 (32.3%)	
1p/19q codeletion status, n (%)			< 0.001
Codel	93 (16.4%)	42 (7.4%)	
Non-codel	161 (28.4%)	270 (47.7%)	
Age, median (IQR)	41 (34, 49.25)	45 (37, 56)	< 0.001

CI confidence interval, IDH isocitrate dehydrogenase, IQR interquartile range.

### 3.2 Association of EDEM2 expression with clinicopathological features

**Figure 1:**
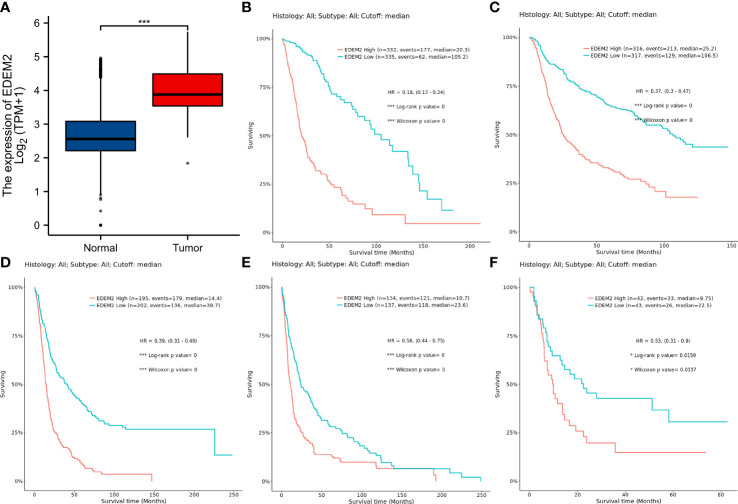
Differential expression analysis of EDEM2 versus normal tissues and Kaplan-Meier curves for multiple datasets pertaining to OS. **(A)** EDEM2 expression in gliomas as determined by TCGA and the GTEx databases. **(B)** TCGA-GBMLGG dataset. **(C)** CGGA (Primary) dataset. **(D)** Rembrandt dataset. **(E)** Gravendeel dataset. **(F)** Freije dataset. * p< 0.05; ***p < 0.001.

**Figure 2:**
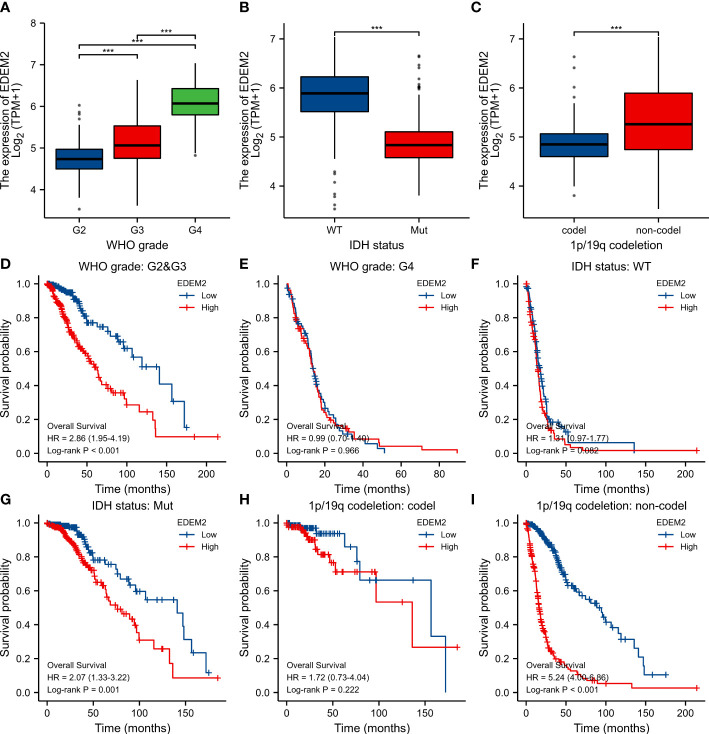
Differential expression and survival analysis of subgroups in TCGA cohort. **(A-C)** EDEM2 expression differences in different pathological conditions, respectively. **(D-I)** Survival curves for EDEM2 in different pathological states. ***p < 0.001.

In the glioma samples, compared to the normal tissues, the expression of EDEM2 was considerably increased (p < 0.001) (**Figure 1A**). The EDEM2 expression was linked to overall survival (OS) in the WHO grade, 1p/19q codeletion, and IDH status, according to subgroup analysis (**Figures 2A–C**). Additionally, the Kaplan-Meier survival analysis showed that in various cohorts, high EDEM2 expression was more significantly linked with a poor prognosis than low EDEM2 expression (**Figures 1B–F**, p < 0.05). In states encoded by LGG, IDH mutation, and 1p/19q codeletion, survival studies under various clinical situations found that increased expression of EDEM2 was linked to a poorer prognosis (**Figures 2D–I**). However, the CGGA cohort revealed that, except in the case of GBM (p = 0.127), EDEM2 expression levels were related to prognosis. This is illustrated in **Supplementary Figure S1**. A relatively higher expression of EDEM2 was found in common cancers, and the prognostic value of EDEM2 in glioma was significantly higher than that in other cancers (**Supplementary Figure S4**).

### 3.3 The relationship between EDEM2 expression and clinical outcomes

**Figure 3:**
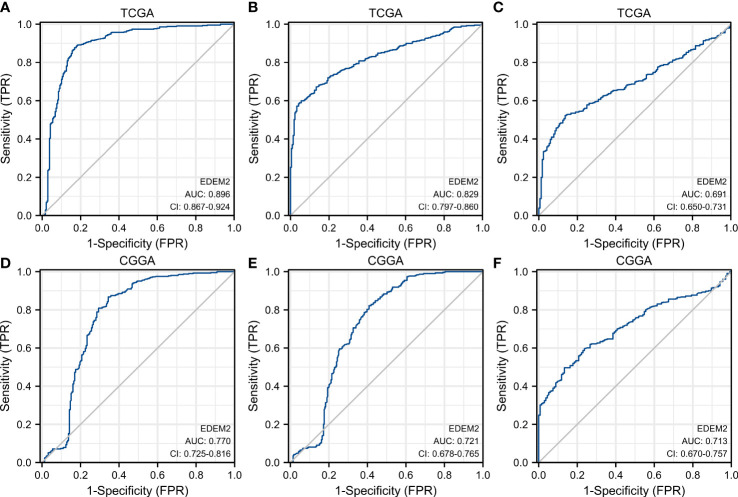
ROC curve to test the value of EDEM2 to identify different pathological features. **(A–C)** WHO grade, IDH status, 1p/19q status in TCGA. **(D–F)** WHO grade, IDH status, 1p/19q status in the CGGA.

Poor survival was found to be substantially correlated with the grade, age, IDH status, 1p/19q codeletion, and EDEM2, as demonstrated in **Table 2**, based on the univariate Cox regression. A multivariate Cox regression analysis was conducted to further investigate the contributing components. It revealed that high EDEM2 expression remained an independent factor linked to poor OS (p = 0.004). EDEM2 was discovered to be an additional independent prognostic factor through a Cox analysis of the validation set (p = 0.034). **Supplementary Table 1** displays the specific outcomes. **Figure 3** displays the ROC curves for the prediction of EDEM2 expression related to pathogenic factors in the two datasets. The area under the curve (AUC) values of the pathological grade curves were the largest, followed by the IDH status, and the smallest for the 1p/19q status.

**Table 2:** Univariate and multivariate Cox analysis results in the TCGA cohort.

Characteristics	Total (N)	Univariate analysis		Multivariate analysis	
		Hazard ratio (95% CI)	P value	Hazard ratio (95% CI)	P value
Age	669				
<=60	530	Reference			
>60	139	4.716 (3.609-6.161)	<0.001	1.584 (1.153-2.174)	0.004
Gender	669				
Female	283	Reference			
Male	386	1.230 (0.955-1.585)	0.109		
WHO grade	612				
G2&G3	452	Reference			
G4	160	9.504 (7.162-12.611)	<0.001	2.320 (1.609-3.345)	<0.001
IDH status	660				
WT	237	Reference			
Mut	423	0.102 (0.077-0.135)	<0.001	0.279 (0.180-0.434)	<0.001
1p/19q codeletion	663				
codel	167	Reference			
non-codel	496	4.635 (2.963-7.251)	<0.001	1.466 (0.866-2.481)	0.154
EDEM2	669				
Low	334	Reference			
High	335	5.946 (4.408-8.020)	<0.001	1.881 (1.228-2.883)	0.004

CI, confidence interval; IDH, isocitrate dehydrogenase; IQR, interquartile range.

### 3.4 Nomogram construction and validation

**Figure 4:**
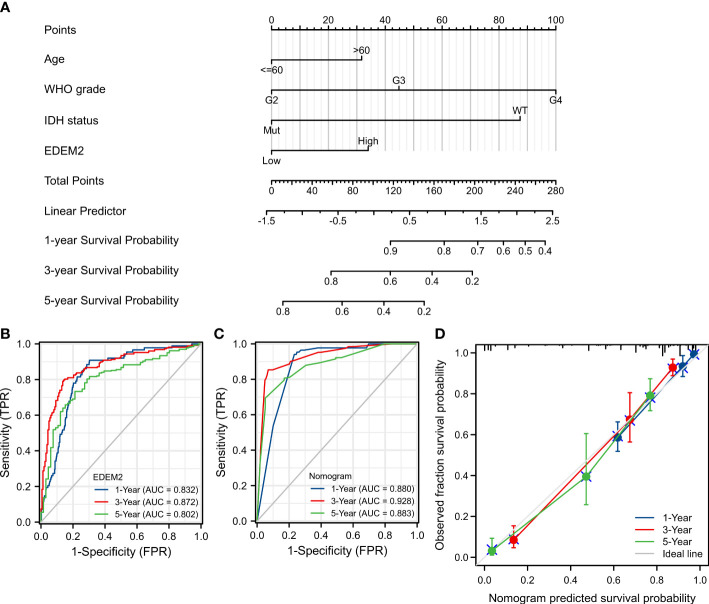
Nomogram establishment and evaluation in TCGA cohort. **(A)** Nomogram based on multivariate Cox analysis. **(B)** Time-dependent ROC curve of EDEM2. **(C)** Time-dependent ROC curve of the nomogram. **(D)** Model calibration curve plot.

A nomogram was constructed using EDEM2 and independent clinical risk factors to provide a quantitative method to predict the prognosis (**Figure 4A**). All variables met the assumption of proportional hazard. The probability of one-, three-, and five-year survival for glioma patients was determined by drawing a vertical line from the total points axis down to the outcome axis. The time-dependent ROC of EDEM2 is shown in **Figure 4B**, and its AUC values are 0.832, 0.872, and 0.802, respectively. Meanwhile, the prognostic model similarly had higher AUC values, i.e., 0.880, 0.928, and 0.883 (**Figure 4C**). As seen in **Supplementary Figure 2**, EDEM2 showed moderate potency, despite having relatively lower AUC values in the validation set (0.636, 0.716, and 0.704), and the AUC value of the test set prediction model was comparable to that of the test set. A C-index of 0.847 (0.836–0.859) was also displayed for the nomogram’s predictive validity. The calibration plot’s deviance was almost 45 degrees close to the ideal curve, suggesting that the anticipated and observed values agreed well (**Figure 4D**).

### 3.5 DEGs functional analysis

**Figure 5:**
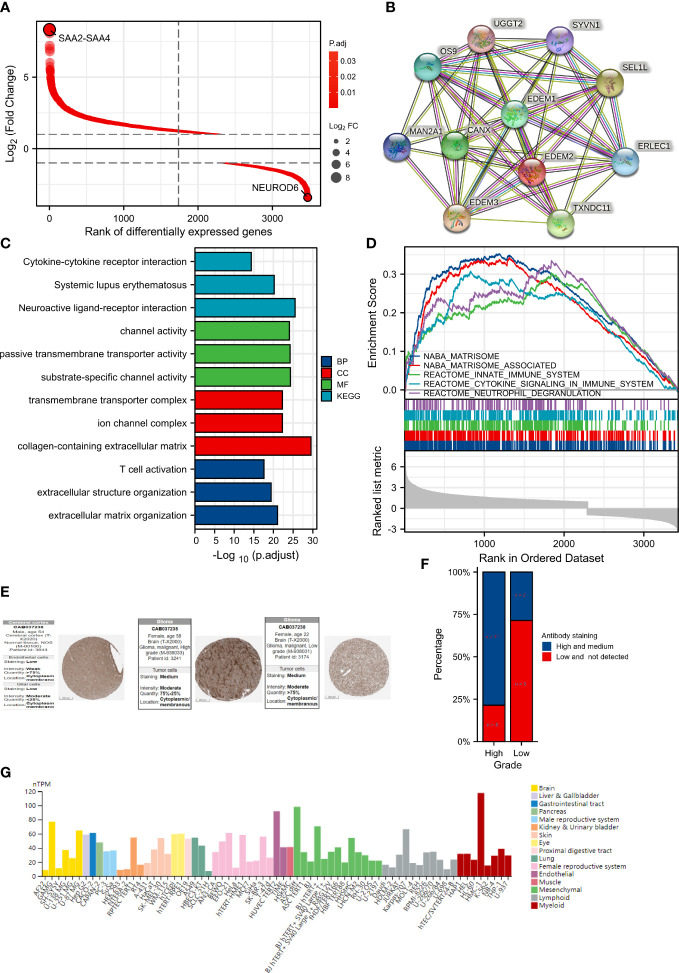
Differential genes (DEGs) analysis between different expression groups and protein expression analysis of EDEM2. **(A)** DEGs Ranking Plot. **(B)** EDEM2 protein interaction network. **(C)** The results of the GO and KEGG analyses. **(D)** GSEA enrichment analysis results. **(E)** Immunohistochemical images of pathological and normal tissues on the HPA sites of varying grades. **(F)** Bar plot of EDEM2 protein expression of glioma samples in the HPA website. **(G)** Expression of EDEM2 in cell lines.

A total of 3474 DEGs were found after comparing the data from two groups, with the high-expression group containing 1155 downregulated genes and 2319 upregulated genes (**Figure 5A**). The relative expression values of the top two DEGs were SAA2-SAA4 and NEUROD6. Protein-protein interactions are crucial to the molecular processes and metabolism of malignant tumors. **Figure 5B** contains a list of the proteins and matching genes related to EDEM2. These include excision repair cross complementation group 1 (ERLEC1), CANX, ER degradation-enhancing alpha-mannosidase–like 1 protein (EDEM1), mannosidase alpha class 2A member 1 (MAN2A1), TXNDC11, ER degradation-enhancing alpha-mannosidase–like 3 protein (EDEM3), Amplified In Osteosarcoma 9 (OS9), suppressor/enhancer of lin-12-like (Sel1L), uridine diphosphate-glucose glycoprotein glucosyltransferase 2 (UGGT2), and synoviolin 1 (SYVN1). The top three considerably enriched items in GO and KEGG were then shown separately for each of the aforementioned four features, as seen in **Figure 5C**. The modulation of extracellular matrix architecture, extracellular matrix including collagen, substrate-specific channel activity, and neuroactive ligand-receptor interaction were the four most substantially enriched characteristics. EDEM2 expression in glioma tissues was confirmed by immunohistochemistry at the HPA locations, and the percentage of highly expressed EDEM2 in these high-grade pathological tissues was rather high (**Figures 5E, F**). EDEM2 expression was also present in the glioma cell lines U251, U87, and U138, with the highest degree of expression in U87 (**Figure 5G**). Additionally, we looked into the EDEM2 gene using the HPA tool’s gene functional enrichment module and discovered that it was rich in the immunological phenotype (**Supplementary Figure 3**).

### 3.6 GSEA identifies EDEM2-related signaling pathways

Significant variations in enrichment discovered by the GSEA were found in the Molecular Signatures Database (MSigDB) Collection. The enhanced pathways were significant and ranked in the top five by the nuclear export signal (NES) value for visualization and presentation, as shown in **Figure 5D**. The five components were matrisome, innate immunity, cytokine signaling in the immune system, neutrophil degranulation, and the cell cycle.

### 3.7 Infiltrating immune cells

**Figure 6:**
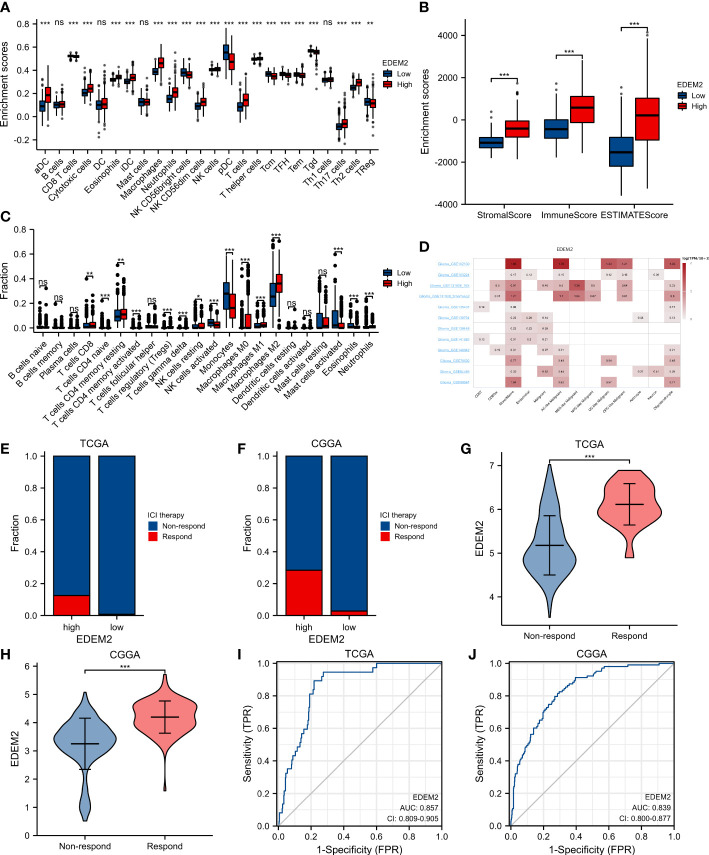
Analysis of immune infiltration and analysis of responsiveness to ICI treatment. **(A)** Immune infiltration by ssGSEA algorithm. **(B)** Immune infiltration by ESTIMATE algorithm. **(C)** CIBERSORT algorithm occupancy of immune cells. **(D)** Results of single-cell transcriptome analysis of EDEM2 based on the TISCH website. **(E, F)** ICI therapy responder distribution in different EDEM2 subgroups. **(G, H)** EDEM2 differences between responders and non-responders in the CGGA and TCGA cohorts. **(I, J)** ROC curve analysis of EDEM2 predicts ICI treatment response in the CGGA and TCGA cohorts. ns, p≥0.05; *p< 0.05; **p<0.01; ***p<0.001.

In cancer patients, tumor-infiltrating lymphocytes independently predict OS and the status of sentinel lymph nodes. We evaluated the relationship between EDEM2 expression and the degree of immune infiltration using the ssGSEA program. Additionally, we discovered that the levels of macrophage, total T lymphocytes (T cells), and neutrophil infiltration were significantly higher in the high-expression group than in the low-expression group, where the proportion of M2 macrophages was the most obvious when considering the differences between various immune infiltration algorithms in combination with the analysis results of CIBERSORT, as shown in **Figures 6A, C**. There was no distinction in the infiltration of dendritic cells (DC) and B lymphocytes (B cells) between the two groups. Further investigation showed that, in comparison to EDEM2-low patients, EDEM2-high patients had considerably greater immune, stromal, and estimated fractions (**Figure 6B**). According to pertinent cohort studies using glioma single-cell transcriptome sequencing, EDEM2 was also abundantly expressed in monocytic macrophages (**Figure 6D**).

### 3.8 Association between EDEM2 expression and ICI therapy outcomes

In both datasets, we found that the EDEM2 expression in the high-expression group was higher than that in the low-expression group based on the TIDE algorithm for the evaluation of potential responses to ICI treatment. The EDEM2 expression in the ICI treatment-response group was significantly higher than that in the non-response group (**Figures 6E–H**). **Figures 6I, J** demonstrate how the ROC curves further assessed the diagnostic effectiveness of EDEM2 on the reactivity to ICI treatment results, with the AUC values for the 2 datasets being 0.857 and 0.839, respectively.

### 3.9 Mutational landscape differences

**Figure 7:**
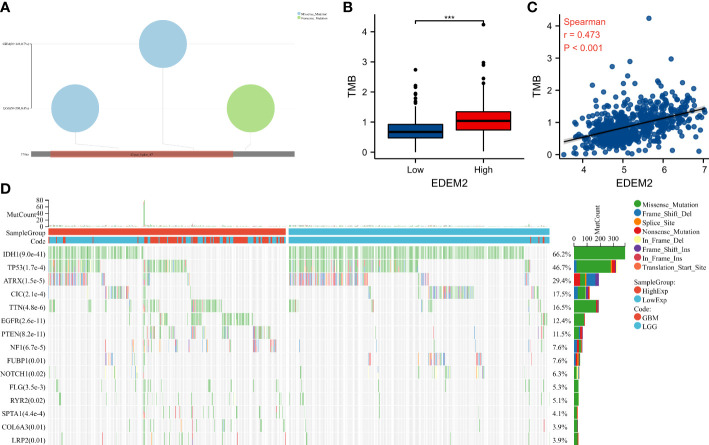
The mutation landscape and tumor mutation burden (TMB) of different EDEM2 expressions in the TCGA cohort. **(A)** Mutation profile in EDEM2. **(B)** Comparison of TMB between the two groups. **(C)** The correlation of TMB scores with EDEM2. **(D)** Differences in gene mutations between the two groups. ***p < 0.001.

The frequency of mutations in the EDEM2 gene in TCGA glioma samples was very low, with 0.4% and 0.7% in LGG and GBM, respectively (**Figure 7A**). The TMB score of the high-expression group was significantly higher than that of the low-expression group (**Figure 7B**). Simultaneously, there was a positive correlation (r = 0.473, p < 0.001) between the TMB score and EDEM2 expression (**Figure 7C**). **Figure 7D** shows the 15 genes with higher mutation frequencies between the 2 groups, with deletion mutations being the most significant. These genes included IDH1, tumor protein 53 (TP53), alpha-thalassemia/mental retardation, X-linked (ATRX); capicua transcriptional repressor (CIC), titin (TTN), epidermal growth factor receptor (EGFR), phosphatase and tensin homolog (PTEN), neurofibromin 1 (NF1), far upstream element-binding protein 1 (FUBP1), neurogenic locus notch homolog protein 1 (NOTCH1), filaggrin (FLG), ryanodine receptor 2 (RYR2), spectrin alpha, erythrocytic 1 (SPTA1); alpha-3 chain of type VI collagen (COL6A3), and low-density lipoprotein-related protein 2 (LRP2).

### 3.10 Validation of EDEM2 expression and immune cell infiltration in clinical samples

**Figure 8:**
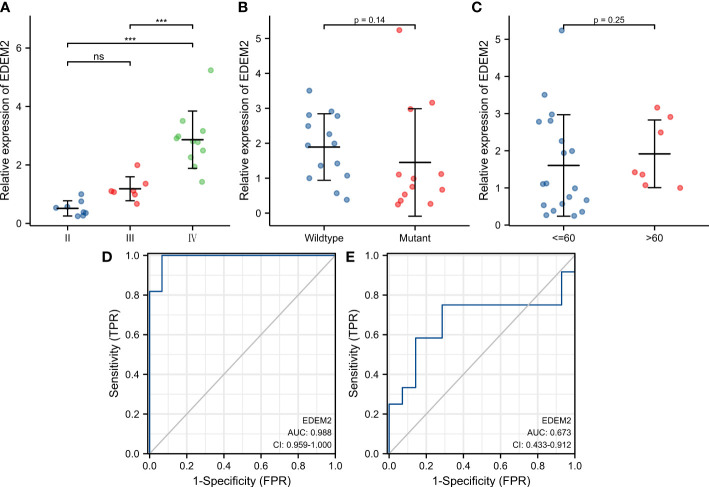
Expression pattern of EDEM2 in pathological specimens. **(A-C)** Pathological grade, IDH status, and age-related differences in EDEM2 expression. **(D)** EDEM2 prediction grade ROC curve. **(E)** EDEM2 ROC curve for IDH status prediction.

**Figure 9:**
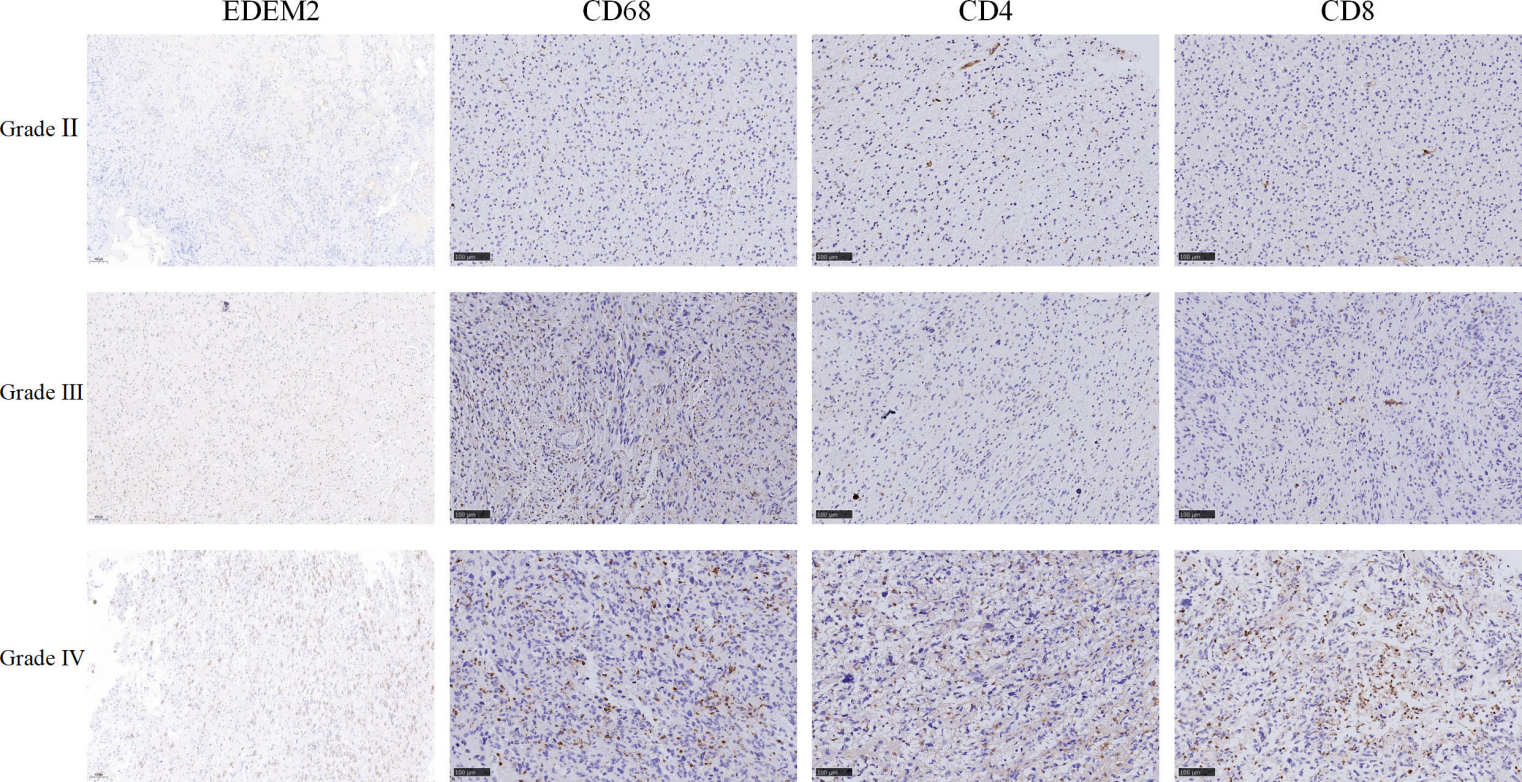
Immunohistochemical picture of three different grades of clinical samples.

**Supplementary Table 2** shows the clinical data of the 26 glioma tissue samples, which include 8 grade-II specimens, 6 grade-III specimens, and 12 grade-IV specimens. As shown in **Figures 8A–C**, there was no discernible variation in the expression of EDEM2 across a range of glioma tissue grades, IDH statuses, or ages. The AUC values of EDEM2 for pathological grade and IDH status were 0.988 and 0.673, respectively, when we additionally drew the ROC curve to assess the diagnostic usefulness of EDEM2 (**Figures 8D, E**). High-grade gliomas had stronger EDEM2 expression based on immunohistochemistry, and the expression of cluster of differentiation 68 (CD68), cluster of differentiation 4 (CD4), and cluster of differentiation 8 (CD8) was higher in the samples (**Figure 9**).

### 3.11 Knockdown of EDEM2 inhibited the invasion and migration of glioma cells

**Figure 10:**
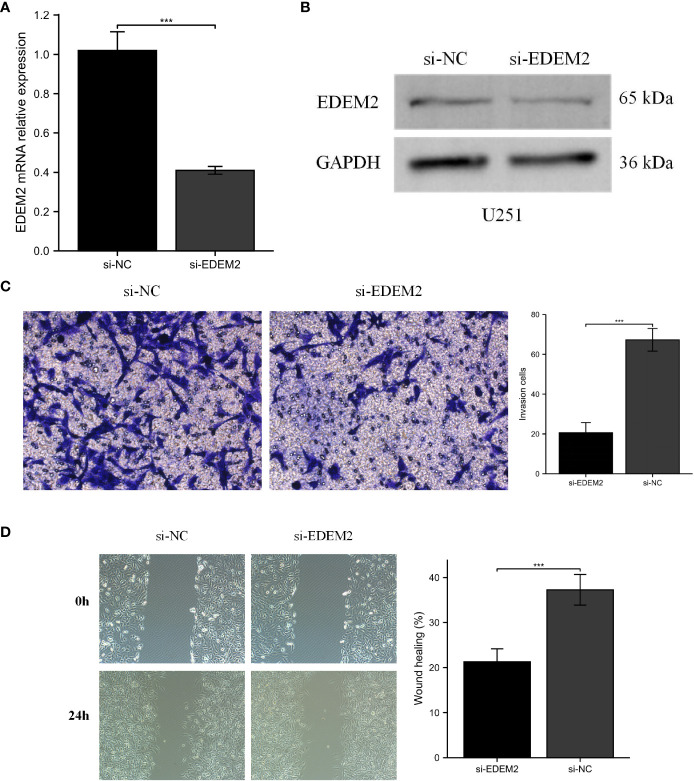
Cell experiments of EDEM2 knockdown. **(A, B)** qPCR and Western blot verified the knockdown status of EDEM2. **(C, D)** the migration and invasion abilities of U251 cells were inhibited by wound healing and transwell assays. ***, P < 0.001.

PCR and Western blot experiments confirmed the knockdown of EDEM2 in U251 cells (**Figures 10A, B**). We evaluated the invasion status of U251 based on wound healing and the transwell assays. As shown in **Figure 10C**, the transwell assay showed that the invasion ability of U251 cells was inhibited by the EDEM2 knockdown group. Similarly, the wound healing rate was significantly decreased in the EDEM2 knockdown group (**Figure 10D**).

### 3.12 Drug prediction and theoretical validation

**Figure 11:**
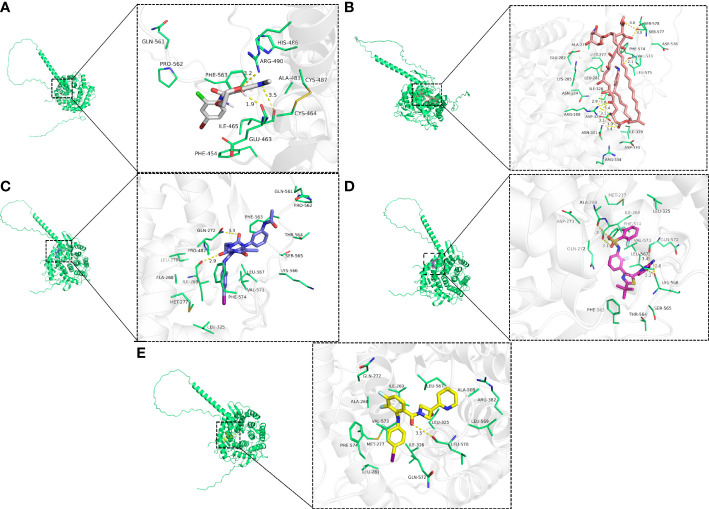
The three-dimensional view of drug-sensitive and EDEM2 docking. **(A)** Selumetinib. **(B)** Mepartricin. **(C)** Trametinib. **(D)** Dabrafenib. **(E)** Cobimetinib.

**Figure 12:**
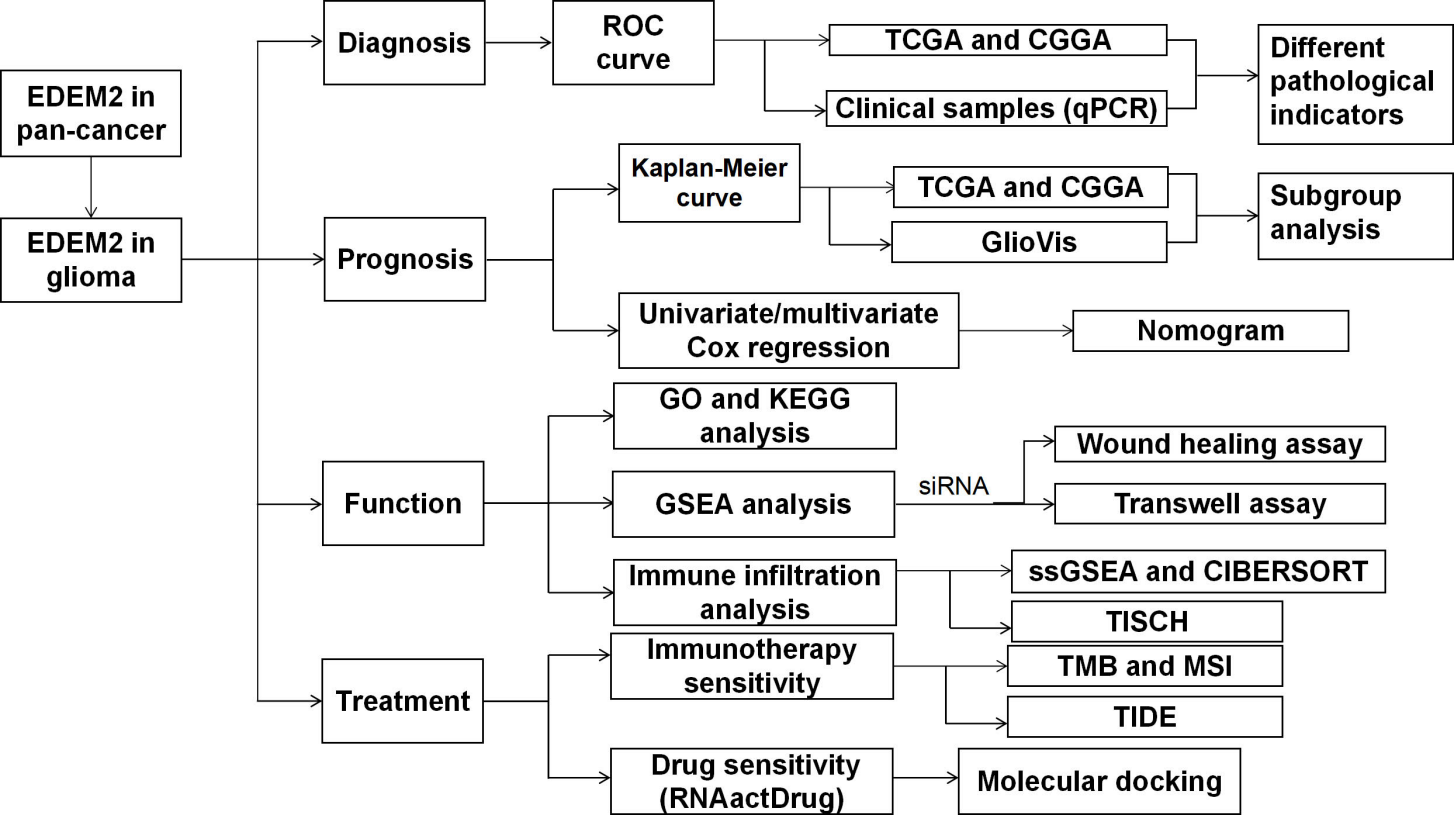
Flow chart.

The top five drugs were identified through a thorough investigation of drug sensitivity utilizing the RNAactDrug website and EDEM2 expression. These were mepartricin, cobimetinib, selumetinib, trametinib, and dabrafenib. We performed molecular docking simulations based on the aforementioned data and found that the binding energy induced by these five drugs to the EDEM2 protein was -8.0,-9.0,-6.1,-7.5, and -8.1 kcal•mol^-1^, demonstrating that all of these substances could stably connect to EDEM2. As illustrated in **Figure 11**, we used PyMOL to examine the interactions between the drugs and the ligand-receptor protein complex. All the drugs could bind to certain amino acid sites in the EDEM2 protein to form hydrogen bonds that would increase the protein’s stability. For a better understanding of this, we drew a flow chart of the entire study (**Figure 12**).

## 4 Discussion

A prognostic biomarker is significant because it offers crucial information about the clinical course and aggressiveness of a patient’s cancer in the absence of therapy, which forms the basis for individualized care (20, 21). A set of genes involved in the ERAD of improperly folded glycoproteins includes EDEM2 (10). As far as our knowledge goes, no study so far examined EDEM2 expression and its possible effects on cases of glioma. Therefore, this study primarily focused on the function of EDEM2 in cases of glioma. We conducted a retrospective study of patients with glioma using data from TCGA, the CGGA, and GlioVis. According to our research, EDEM2 expression is a reliable indicator of a glioma patient’s prognosis and plays a role in controlling the tumor’s immune microenvironment.

The ERAD pathway, which targets misfolded glycoproteins for degradation *via* the proteasome in an N-glycan-dependent manner, involves the EDEM family of proteins (22–24). Its precise molecular process in the development of tumors remains unknown. The diagnostic and prognostic utility of EDEM2 in glioma was examined in this study using high-throughput RNA sequencing data for integrated bioinformatics analysis. In contrast to normal samples, we discovered that the expression of EDEM2 was greater in tumor tissues. Furthermore, high-grade, IDH wild-type, and 1p/19q non-coding tumor tissues had significant levels of EDEM2 expression. Additionally, all of the ROC curves for the diseased components mentioned above had modest AUC values, suggesting that EDEM2 may be used as a marker to aid pathological diagnosis. The aforementioned findings were similarly supported by a real-world expression investigation of EDEM2. Furthermore, we discovered a strong correlation between high EDEM2 expression and worse prognostic outcomes, with good predictive potential. We did not include recurrence samples, as was the case with other single-gene studies (25, 26), and we validated the prognostic results using a large data collection. The Cox regression-based nomogram also showed strong predictive power. Thus, EDEM2 may be used as a possible biomarker to determine a patient’s diagnosis and prognosis in cases of glioma.

We conducted GO/KEGG and GSEA analyses to investigate the function of EDEM2 in GBM in more detail. According to the findings of the functional enrichment analysis of DEGs, EDEM2 may have a role to play in the control of extracellular matrix and membrane signaling in gliomas. These findings are consistent with how EDEM2 is known to work. First, EDEM2 controls the glycoprotein modification and breakdown, and several glycoproteins are essential for intercellular communication (27). Second, we propose that a block in tumor cells may have been caused by the ERAD process, which is mediated by EDEM2 (6, 27, 28). Current research has shown that glioma cells frequently activate the perk arm of the UPR by increasing the levels of the metalloproteinases (MMPs) MMP2 and MMP7, which promote the epithelial-mesenchymal transition (EMT) (29, 30). The association of EDEM2 with extracellular matrix changes was further corroborated by the observation that EDEM2 knockdown suppressed glioma cell invasion. Furthermore, it increased the expression of the gene for the lysosomal-associated membrane glycoprotein 3 (LAMP3), which stimulated cell adhesion and aided in the development of cellular filopodia (31). The PPI network of EDEM2’s connexins revealed that every connexin was engaged in the ERAD process (7, 32–34). This highlighted the possibility that EDEM2 overexpression could have been responsible for activating the ERAD/UPR pathway (22). Further research is still required to fully understand the intricate molecular processes as well as the more intricate regulatory networks because of the intricacy of UPR-related pathways.

Immune cells that invade tumors play a crucial role in the growth and evolution of tumors, and it is assumed that the distribution and makeup of these cells affect the prognosis of a certain kind of tumor (35). The GSEA analysis revealed that EDEM2 may be involved in the control of the immunological microenvironment, particularly in the innate immune system. The immune infiltration investigation supported our conclusions. In comparison to the EDEM2-low group, the immunological, stromal, and estimated fractions were higher in the EDEM2-high group. Further investigation revealed that increased neutrophils and macrophages, particularly those of the M2 type, were mostly associated with the high expression of EDEM2. The findings of single-cell transcriptome-based studies also showed that monocytic macrophages expressed EDEM2 to various degrees. The M2-type macrophages aid in the development of an immunosuppressive microenvironment, which impedes the immune system’s ability to destroy tumor cells and, as a result, worsens the prognosis (36, 37). According to studies, the IRE1-dependent X-Box binding protein (XBP1) signaling pathway during UPR has been demonstrated to support angiogenesis and immune cell infiltration of tumors (8, 38). Hence, immune cell infiltration may have been influenced indirectly by the UPR brought on by EDEM2. In recent years, ICI treatment response has been routinely predicted using the TIDE computational technique (17). This is why we used the TIDE method to predict how well various groups respond to ICI treatment. The high-expression group in both cohorts had a higher percentage of patients who responded to ICI therapy than the low-expression group. The results of additional research showed that the respondents expressed more EDEM2 than non-responders. In comparison to several recent multi-gene model studies, ROC analysis demonstrated that EDEM2 has a high degree of discriminating capacity in response to ICI therapy (33). Therefore, we hypothesized that by modifying the immune infiltrate in gliomas, EDEM2 would affect patient prognosis.

Tumor incidence and development often entail gene mutations. IDH and tumor protein 53 (TP53) are two examples of frequently-occurring gene alterations in cases of glioma (39). We discovered that EDEM2 mutations in gliomas were rare. We also contrasted the variations in gene mutations across various expression groups. The IDH1 gene had the highest mutation frequency, whereas the EDEM2 high-expression group had much lower mutation rates than the low-expression group. It is widely known that the prognosis of IDH mutant glioma is better than that of IDH wild-type glioma (2, 40). This may be a contributing factor in the bad prognosis of EDEM2 overexpression. In the therapy of ICI, TMB emerged as a new possible biomarker that is closely correlated with the number of new antigens generated in TME (41). This study demonstrated that the high-expression EDEM2 group with increased TMB may respond more favorably to ICI therapy. Finally, we performed a drug sensitivity analysis and molecular docking to investigate possible therapeutic medications (27). We discovered five substantially related pharmaceuticals, including one antifungal agent and four molecularly targeted therapies. Therefore, our findings offer a fresh perspective on treating targeted EDEM2.

Our study has some limitations, despite providing a better understanding of the connection between EDEM2 and glioma. Retrospective research has its own limits, even though it entails multicenter results in open databases. Prospective investigations should be conducted in the future to prevent analytical bias. Second, we could only evaluate the predictive value of EDEM2 for ICI treatment by employing cohorts of metastatic melanoma and urothelial carcinoma because of the limits of the TIDE algorithm. The biological role of EDEM2 in glioma must be demonstrated through more experimental confirmation.

In glioma patients, elevated EDEM2 expression was shown to be substantially related to a bad prognosis for the first time in our investigation, which suggests that it may encourage carcinogenesis through abnormal immune responses. Our findings imply that EDEM2 may be useful as a biomarker for the diagnosis, treatment, and prognosis of gliomas. However, more research is required to confirm this. Nomograms were also developed for a case-specific and thorough examination. Our study certainly provides fresh perspectives that would enable a deeper understanding of the molecular etiology of EDEM2.

## Data availability statement

The original contributions presented in the study are included in the article/ **Supplementary Material**. Further inquiries can be directed to the corresponding author.

## Ethics statement

Written informed consent was obtained from the individual(s) for the publication of any potentially identifiable images or data included in this article.

## Author contributions

The authors confirm contribution to the paper as follows: study conception and design: YW, HW. Data collection: YW, HW. Analysis and interpretation of results: YW, DY, WX. Draft manuscript preparation: YW, DY, WX. All authors contributed to the article and approved the submitted version.

## Supplementary material

Supplementary Figure 1 Differential expression and survival analysis of subgroups in the CGGA cohort. (A-C) EDEM2 expression differences in different pathological conditions, respectively. (D-I) Survival curves of EDEM2 in different pathological statuses, respectively. ***p < 0.001.

Supplementary Figure 2 Model efficacy evaluation of the CGGA cohort. (B) Time-dependent ROC curve of EDEM2. (C) Time-dependent ROC curve model.

Supplementary Figure 3 Functional enrichment of EDEM2 in the HPA website. (A) Cell lines level. (B) Single-cell level.

Supplementary Figure 4 EDEM2 in pan-cancer. (A) Expression analysis. (B) Survival analysis.
