# Supplementary figures and images for: EDEM2 is a diagnostic and prognostic biomarker and associated with immune infiltration in glioma: A comprehensive analysis
Source: Front Oncol. 2023 Jan 16;12:1054012. doi: 10.3389/fonc.2022.1054012 (PMC9885217; doi:10.3389/fonc.2022.1054012)

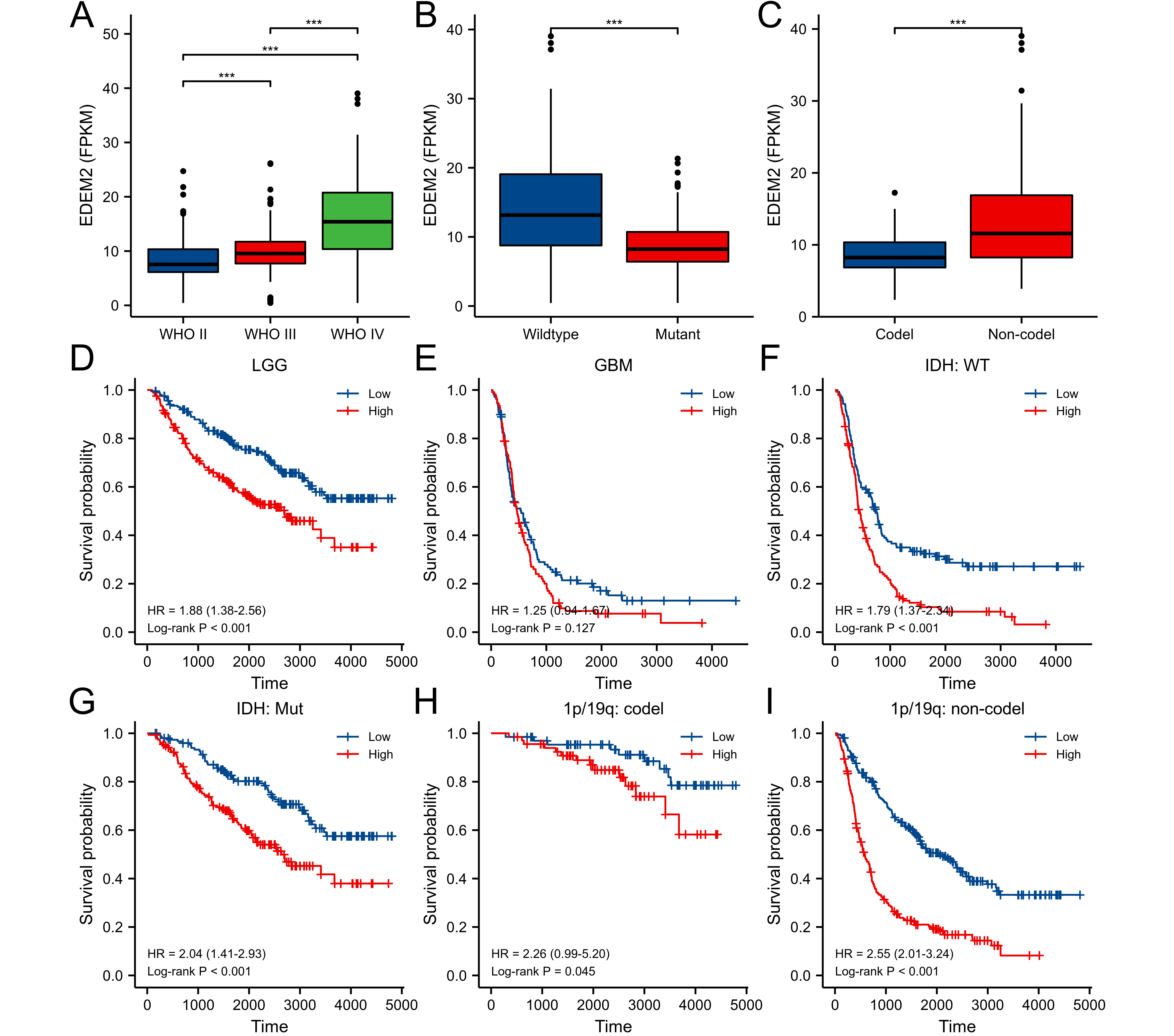

Supplement: Supplementary Figure 1 — Differential expression and survival analysis of subgroups in the CGGA cohort. (A-C) EDEM2 expression differences in different pathological conditions, respectively. (D-I) Survival curves of EDEM2 in different pathological statuses, respectively. ***p < 0.001. [file Image_1.tiff]

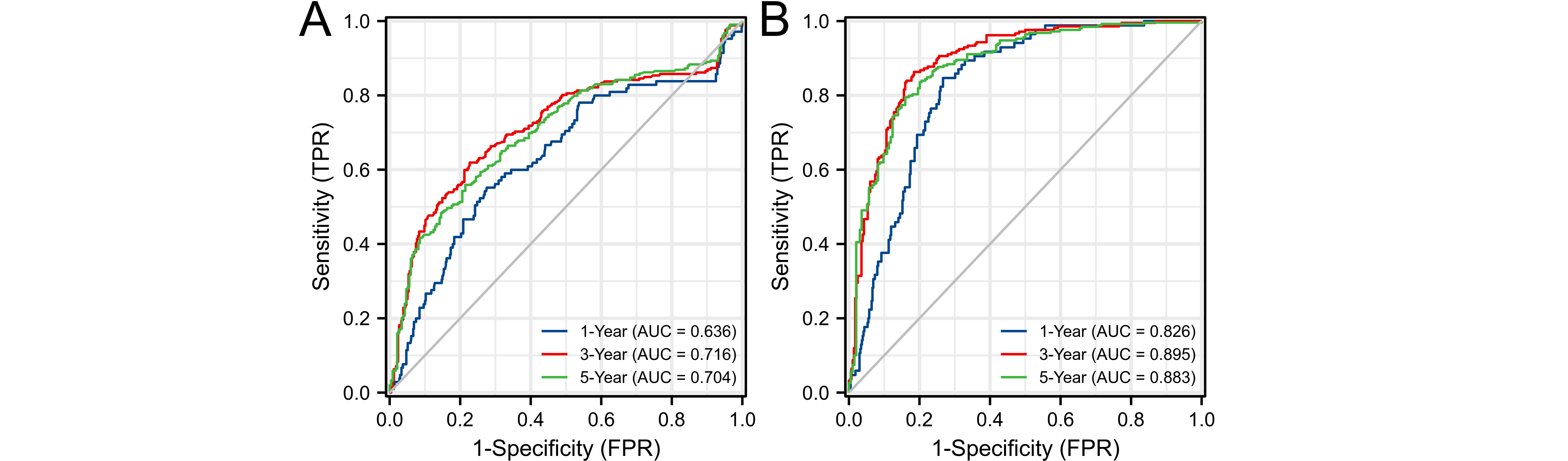

Supplement: Supplementary Figure 2 — Model efficacy evaluation of the CGGA cohort. (B) Time-dependent ROC curve of EDEM2. (C) Time-dependent ROC curve model. [file Image_2.tiff]

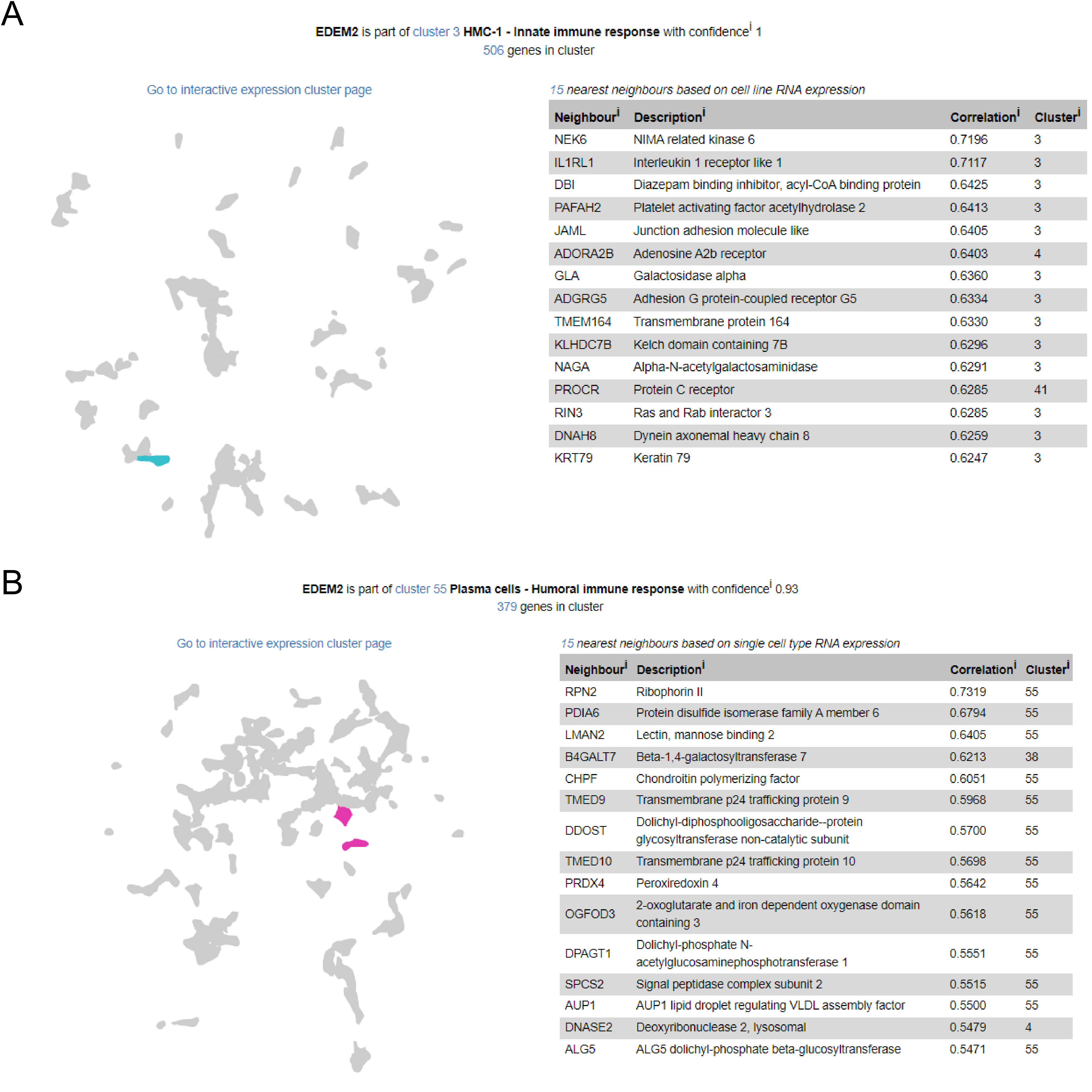

Supplement: Supplementary Figure 3 — Functional enrichment of EDEM2 in the HPA website. (A) Cell lines level. (B) Single-cell level. [file Image_3.tiff]

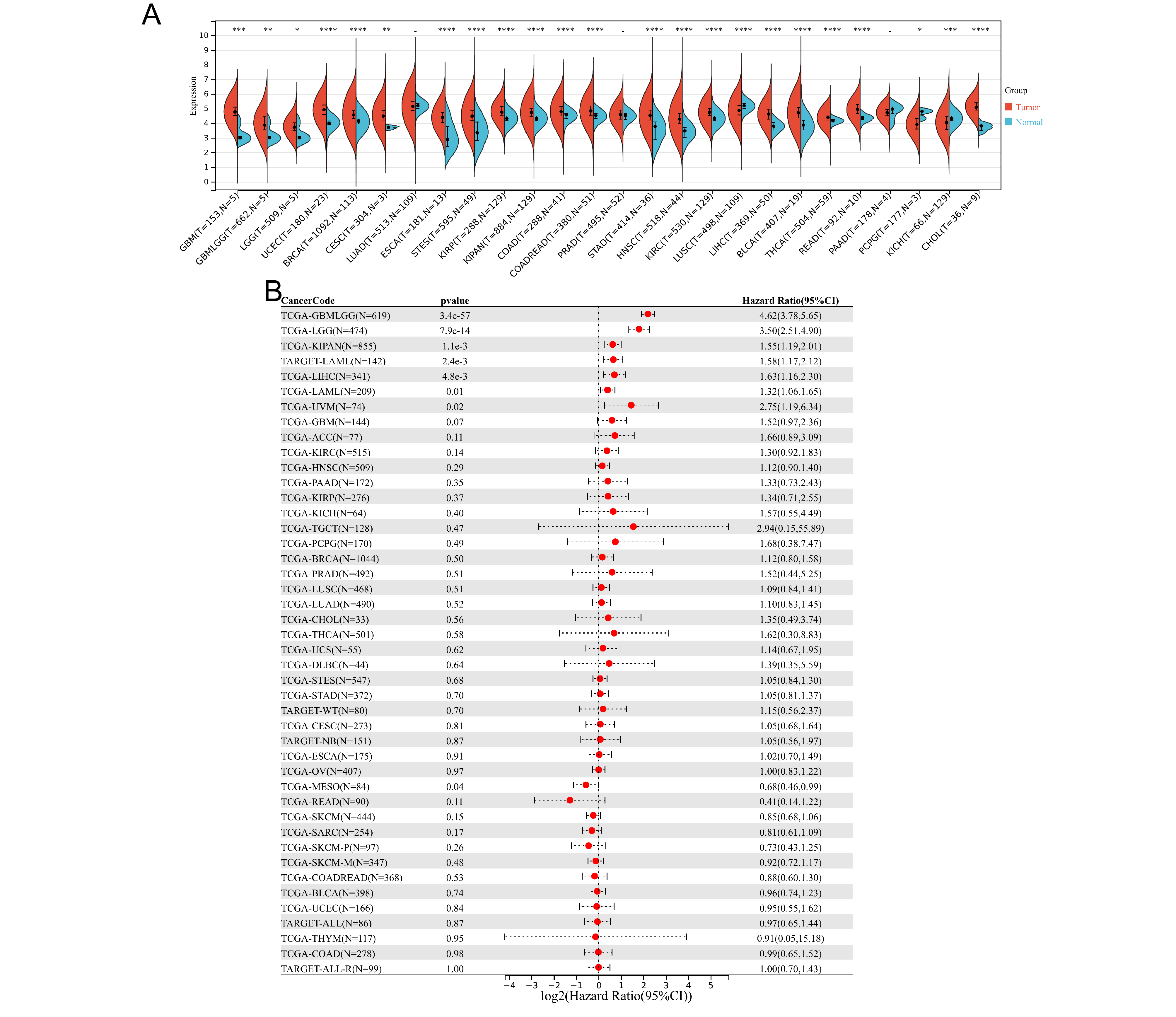

Supplement: Supplementary Figure 4 — EDEM2 in pan-cancer. (A) Expression analysis. (B) Survival analysis. [file Image_4.tiff]
