# Supplementary material for: EDEM2 is a diagnostic and prognostic biomarker and associated with immune infiltration in glioma: A comprehensive analysis
Source: Front Oncol. 2023 Jan 16;12:1054012. doi: 10.3389/fonc.2022.1054012 (PMC9885217; doi:10.3389/fonc.2022.1054012)
Supplement: Supplementary file 5 [file Table_1.docx]

**Supplementary Table 1.** Cox regression analysis results for the CGGA dataset.

| Characteristics | Total(N) | Univariate analysis | |  | Multivariate analysis | |
| --- | --- | --- | --- | --- | --- | --- |
|  |  | Hazard ratio (95% CI) | P value |  | Hazard ratio (95% CI) | P value |
| IDH mutation status | 588 |  |  |  |  |  |
| Wildtype | 279 | Reference |  |  |  |  |
| Mutant | 309 | 0.222 (0.176-0.280) | <0.001 |  | 0.620 (0.460-0.835) | 0.002 |
| 1p/19q codeletion status | 566 |  |  |  |  |  |
| Non-codel | 431 | Reference |  |  |  |  |
| Codel | 135 | 0.139 (0.091-0.213) | <0.001 |  | 0.265 (0.166-0.424) | <0.001 |
| Grade | 626 |  |  |  |  |  |
| WHO IV | 218 | Reference |  |  |  |  |
| WHO III | 188 | 0.299 (0.233-0.384) | <0.001 |  | 0.710 (0.514-0.981) | 0.038 |
| WHO II | 220 | 0.117 (0.087-0.158) | <0.001 |  | 0.278 (0.189-0.410) | <0.001 |
| Gender | 626 |  |  |  |  |  |
| Male | 369 | Reference |  |  |  |  |
| Female | 257 | 0.934 (0.753-1.160) | 0.539 |  |  |  |
| Age (IQR) | 625 | 1.045 (1.035-1.054) | <0.001 |  | 1.017 (1.008-1.027) | <0.001 |
| EDEM2 | 626 | 1.078 (1.063-1.093) | <0.001 |  | 1.020 (1.001-1.039) | 0.034 |

CI confidence interval, IDH isocitrate dehydrogenase, IQR interquartile range.
