# Supplementary material for: EDEM2 is a diagnostic and prognostic biomarker and associated with immune infiltration in glioma: A comprehensive analysis
Source: Front Oncol. 2023 Jan 16;12:1054012. doi: 10.3389/fonc.2022.1054012 (PMC9885217; doi:10.3389/fonc.2022.1054012)
Supplement: Supplementary file 6 [file Table_2.docx]

**Supplementary Table 2.** Clinical features of tissues used for qRT-PCR.

| NO. | Gender | Age | WHO grade | IDH1 status |
| --- | --- | --- | --- | --- |
| 1 | Female | 64 | II | Wildtype |
| 2 | Male | 73 | Ⅳ | Wildtype |
| 3 | Female | 47 | Ⅳ | Wildtype |
| 4 | Male | 56 | III | Mutant |
| 5 | Male | 53 | Ⅳ | Wildtype |
| 6 | Female | 69 | III | Wildtype |
| 7 | Female | 41 | Ⅳ | Wildtype |
| 8 | Male | 70 | Ⅳ | Mutant |
| 9 | Male | 33 | II | Mutant |
| 10 | Male | 43 | II | Mutant |
| 11 | Male | 56 | Ⅳ | Wildtype |
| 12 | Female | 38 | III | Mutant |
| 13 | Male | 39 | II | Mutant |
| 14 | Female | 70 | III | Wildtype |
| 15 | Female | 29 | II | Mutant |
| 16 | Female | 57 | III | Mutant |
| 17 | Female | 45 | II | Wildtype |
| 18 | Female | 50 | III | Mutant |
| 19 | Male | 59 | Ⅳ | Wildtype |
| 20 | Male | 34 | II | Mutant |
| 21 | Male | 27 | II | Wildtype |
| 22 | Female | 27 | Ⅳ | Wildtype |
| 23 | Male | 26 | Ⅳ | Mutant |
| 24 | Male | 67 | Ⅳ | Wildtype |
| 25 | Female | 46 | Ⅳ | Mutant |
| 26 | Male | 74 | Ⅳ | Wildtype |

IDH Isocitrate dehydrogenase.
